# Expenditure Pattern for TB Treatment among Patients Registered in an Urban Government DOTS Program in Chennai City, South India

**DOI:** 10.1155/2012/747924

**Published:** 2012-11-19

**Authors:** Ramya Ananthakrishnan, M. Muniyandi, Anita Jeyaraj, Gopal Palani, B. W. C. Sathiyasekaran

**Affiliations:** ^1^Department of Community Medicine, Sree Balaji Medical College and Hospital, Chennai 600044, India; ^2^National Institute for Research in Tuberculosis, Indian Council of Medical Research, Chennai 600031, India; ^3^Department of Community Medicine, Sree Ramachandra Medical College and Research Institute, Chennai 600116, India

## Abstract

*Background*. Tuberculosis (TB) patients registered in the government clinics under the DOTS (Directly Observed Treatment, Short Course) program in Chennai city catering to about 4.3 million population. *Objective*. To estimate the pattern and overall costs incurred by the new patients (who have never had treatment for tuberculosis or have taken antituberculosis drugs for less than one month) registered under DOTS program in the treatment of tuberculosis in Chennai city. *Methodology*. A cross-sectional survey among new TB patients, who had completed intensive phase of antituberculosis treatment, was done using a precoded semi-structured questionnaire between March and June 2007. Information was collected on demographic, socioeconomic characteristics and expenditure for before and during treatment. Mean costs were used for comparison. *Results*. Among the 300 TB patients, most economically productive age group and 186 (62%) were males. The overall estimated total costs incurred right from the onset of symptoms until treatment completion was found to be Rs. 3211 (3.8% of annual family income) under DOTS program, which is less compared to previous studies. The overall mean total cost was significantly high among male (Rs. 3270; *P* < 0.01), employed (Rs. 3945; *P* < 0.01), and extrapulmonary patients (Rs. 3915; *P* < 0.01). *Conclusion*. The study has reiterated the fact that DOTS helps in reducing out-of-pocket expenses to patients with tuberculosis and hence is a cost-effective health intervention. This cost reduction may help to increase the access to the poor people which would help in achieving universal access to TB care services.

## 1. Introduction

TB (tuberculosis) causes enormous social and economic disruption and hampers nation's development [1, 2]. India accounts for one-fifth of the global TB burden, with 1.8 million developing the disease each year and of them about 800,000 are infectious. Nearly 0.4 million are dying due to TB annually which translates to two deaths every three minutes [3]. The disease is most prevalent in the age group of 15 to 54 years [4, 5], which is the highly economically productive period of an individual's life with important consequences for the household when the individual falls sick with TB.

Generally, burden of TB is measured by morbidity and mortality which are key considerations [6]. However, only focusing on morbidity and mortality effects provides an incomplete picture of the adverse impact of ill health on human welfare. In particular, the economic consequences of poor health can be substantial. Health “shocks,” such as unexpected increases in health expenditure, reduced functional capacity, and lost income or productivity are often a primary risk factor for impoverishment [7, 8]. At a societal level, poor population health is associated with lower savings rates, lower rates of return on capital, and lower levels of domestic and foreign investment; all of these factors can and do contribute to reductions in economic growth [9]. Measurement of these various adverse impacts provides decision-makers to take appropriate policy decisions and also to provide another dimension of justification for worth of investment in TB control [10–12].

Projections from earlier study conducted prior to the implementation of RNTCP (Revised National TB Control Program) in south India indicated that despite being offered free diagnosis and treatment by government, the projected out-of-pocket expenditure incurred by TB patients annually was more than US$ 3 billion [13, 14]. A subsequent study in a rural area showed that for patients registered under RNTCP, the patients who returned to work early established the economic benefits to patients treated under DOTS (Directly Observed treatment, Short Course) [15]. Ten years post-RNTCP implementation, the present study was undertaken to estimate the pattern and overall cost incurred by the patients registered under RNTCP for the treatment of tuberculosis in Chennai city.

## 2. Methodology

### 2.1. Study Setting

Chennai city has a population of 4.34 million and is spread over 174 sq. km. The city is divided into 10 administrative zones with one tuberculosis unit (TU) in each zone. This study has been conducted so as to cover all patients, meeting the eligibility criteria, who come to each TU for DOT in one week of drug cycle.

### 2.2. Study Design and Period

In this cross-sectional study, the data was collected during the period from March 2007 to June 2007.

### 2.3. Study Population

New (who have never had treatment for tuberculosis or have taken antituberculosis drugs for less than one month) adult TB patients of both sexes, coming to the TU for DOT treatment and whose HIV statuses were negative were considered eligible for the study.

### 2.4. Tool for Data Collection

A pretested, semi-structured, precoded questionnaire was used to collect information on demographic and socioeconomic characteristics of patients. The questionnaire also included information on expenditure for the consultation fees, investigations, medicines, travel for escort, and patient before and during treatment. 

### 2.5. Definitions Used

#### 2.5.1. Direct Costs

Consultation fees and money spent on investigations and drugs were classified as medical expenditure. Money spent on travel, lodging, special food, and expenditure incurred for persons accompanying the patient were classified as nonmedical expenditure.

#### 2.5.2. Indirect Costs

Indirect costs were classified as loss of wages due to illness, decreased earning ability due to illness, or long-term disability that necessitated change in type of work. 

#### 2.5.3. Total Cost

Total cost includes the expenditure incurred pretreatment and during treatment under direct and indirect costs.

### 2.6. Data Collection

List of TB patients who met the eligibility criteria was compiled from the TB register. All the patients who met the eligibility criteria were interviewed after the completion of intensive phase of their treatment by trained investigators after obtaining written informed consent either at the TU or in their residence. Patients were informed in their mother tongue, in Tamil or a language that they understood, about the purpose of the study. Patients were told about the confidentiality of the data collected, each interview will take about half an hour to complete it and also it is their right to withdraw from the study at any time. Ethical approval for the study was obtained from the Ethics Committee of Sri Ramachandra University, Porur, Chennai. Costs data were validated throughout the interview by repeated questioning and cross checked with the prevailing rates of doctors consultation fees, costs for investigations, and market price of drugs, medical bills wherever possible. All the information was estimated for 3 periods: (1) before treatment (three months prior to start of TB treatment), (2) intensive phase of treatment (two months treatment period), and (3) continuation phase of treatment (four months after intensive phase). In calculating total treatment costs, the cost was calculated by adding costs of intensive phase and continuation phase for the whole 6 months treatment period. The costs were calculated in terms of Indian rupees and US dollars (exchange rate during the study period US $1 = Rs. 45).

### 2.7. Data Management

Data were checked for errors, entered, and analyzed using the SPSS version 15.0 (SPSS Inc., Chicago, IL, USA). Average (mean and median) costs such as direct medical, direct nonmedical, indirect, and total were calculated for both before and during treatment. All these patients had taken treatment in government hospitals where the investigations and the medicines were offered free of costs. However, they had to spend for travel, and so forth, the distribution of costs is uneven and we are of the opinion that this variation is expected from all economic data such as income and expenditure, and so forth. In order to avoid any bias we have provided both mean and median values. Further we classified the costs into Nil costs, those who spent up to Rs. 500, those who spent more than 500; then we had also measured what proportion were in these categories. Student *t*-test was used to compare the mean difference between mean total costs. A *P* value < 0.05 was considered statistically significant. 

## 3. Results

### 3.1. Coverage

Among the 335 patients who met the eligibility criteria, 305 (92%) were interviewed. About 30 patients could not be interviewed because they had defaulted or migrated. Of 335, only in five patients the costs were quite high as they were out layers and inclusion of this data might distort the average hence excluded from analysis. They had gone to private providers and spent more money for TB diagnostics, test-like scanning, blood test, and so forth, this hiked the total costs and these five were excluded as outliers. Accordingly, further analysis of data relates to 300 patients only. The percentage of coverage in all the ten TUs ranged from 87% to 95%. 

### 3.2. Socioeconomic Profile of the Patients

Majority of the patients (87%) belonged to the less than 54 years age group and the study group included 186 (62%) males and females 114 (38%). Most of the patients 132 (44%) had primary school education and about 114 (38%) were unemployed/retired or were students. Based on monthly percapita income, patients were grouped into three quartiles: less than Rs. 1100 = low income, Rs. 1100 to Rs. 2250 = middle income, and above Rs. 2250 = high income. About 158 (52.7%) had a monthly per-capita income in the range US$ 24.4 to 50 (Table 1).

### 3.3. Overall Direct, Indirect, and Total Costs

The mean direct costs was Rs. 1071 and with a wide range from Rs. 45 to Rs. 8565 (Median Rs. 573). Of this, the mean medical costs was Rs. 765 (range Rs. 18 to Rs. 7818, Median Rs. 353). The mean nonmedical costs were Rs. 306 (range Rs. 27 to Rs. 3377, median Rs. 167). The mean indirect costs was Rs. 2140 (range Rs. 745 to Rs. 18745, median Rs. 745). The mean total cost was Rs. 3211 (range Rs. 790 to Rs. 19360, median Rs. 1615).

### 3.4. Costs according to Patient Characteristics

The overall direct, indirect and total costs according to patients' characteristics is described in Table 1. Overall mean total costs was significantly higher among males (Rs. 3270 versus 2380; *P *< 0.01), employed (Rs. 3945 versus Rs. 2014; *P* < 0.01), those who were living in nuclear family (Rs. 3286 versus Rs. 3025) and among extrapulmonary patients (Rs. 3915 versus Rs. 2951; *P* < 0.01).

The overall mean indirect costs were significantly higher among patients in the age group of less than 54 (Rs. 2215), males (Rs. 2737), those with primary level education (Rs. 2312), employed (Rs. 2935), those with family size of ≤4 (Rs. 2242), those living in nuclear families (Rs. 2217) and among extrapulmonary tuberculosis patients (Rs. 2098).

The overall mean direct costs was higher among patients in the age group of less than 54 (medical Rs. 789 and nonmedical Rs. 312), among females (medical Rs. 855 and nonmedical Rs. 360), those with high school education (medical Rs. 1117 and nonmedical Rs. 306), unemployed (medical Rs. 821 and nonmedical Rs. 351), and extrapulmonary TB patients (medical Rs. 1286 and nonmedical Rs. 531).

### 3.5. Costs Incurred during Different Phases of Treatment

On analyzing the mean costs for the patients during different phase of treatment, the direct cost was Rs. 961 (medical Rs. 743, nonmedical Rs. 219) and it was higher before treatment. The indirect cost was higher during treatment period Rs. 1694 (intensive phase Rs. 949 and continuation phase Rs. 745) (Table 2).

### 3.6. Costs Incurred by Patients prior to Start of Treatment (Pretreatment Costs)

Before starting treatment in RNTCP 81.7% incurred medical costs, 93% incurred direct nonmedical costs and 21% incurred indirect costs (Table 3). During intensive phase of treatment 2.3% incurred medical costs, 42% incurred direct nonmedical costs and 24.7% incurred indirect costs. In all during treatment 45% did not incur any costs (Table 4).

### 3.7. Proportion of Costs to the Annual Family Income

**Figure 1:**
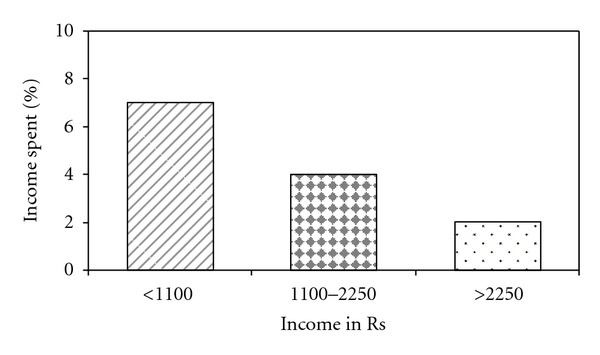
Proportion of income spent for diagnosis and treatment by different income groups.

Proportion of total costs as percentage of annual family income for all 300 patients was 3.8%; the proportion was significantly higher (7%, 4%, 2%; *P* = 0.000) for patients belonging to lower economic strata compared to those in higher economic strata (Figure 1).

## 4. Discussion

This study has highlighted the patterns of expenditure of TB patients in terms of direct costs, indirect costs, and total costs for both diagnosis and treatment by the TB patients registered in government TB control program in Chennai city. The overall estimated total costs incurred right from the onset of symptoms until treatment completion were found to be Rs. 3211 under DOTS program. If these costs were extrapolated to the whole country, the cost to the country for TB treatment would be considerable. However, the encouraging finding is that the estimated mean total cost in the current series was less as against those reported in earlier studies s [9, 16–18]. Our results strongly suggest that DOTS program is cost saving for patients and thus lends support for universal DOTS in India. This finding also draws attention to the contribution tuberculosis control makes to alleviation of poverty by reducing the economic burden that the disease inflicts on the poor.

In this study, we observed that the total costs as percentage of annual family income to the patients was 3.8%. This is much less compared to the earlier study done in rural Tamilnadu; it was in the range from 10 to 19% and most of the study subjects (62%) had per capita income per month <Rs. 335 [11]. This could be due to the fact that this study was done in an urban area where majority of the patients were in monthly percapita income range of more than Rs. 1100. 

In this study, 81.7% and 93% of patients incurred medical and nonmedical costs, respectively, before patients were actually started on treatment in the DOTS programme. In general in more than 50% of chest symptomatics the first point of contact was private health facilities [19–21], these patients had gone to private and spent money for diagnosis which could have been avoided if the private healthcare providers were involved in RNTCP. Therefore, involvement of private health providers is the priority area for RNTCP [22]. Other reason may be due to low awareness among patients who spent more money for “shopping for diagnosis” [23]. advocacy, communication, and social mobilization (ACSM) activities on availability of free TB diagnosis and treatment need to be strengthened to reduce these pretreatment costs.

We also found that less proportion (24.7%) of patients incurred indirect costs as compared to the earlier studies where the indirect costs during treatment was incurred by 54% and 41% of patients, respectively [9, 11]. This finding suggests that due to decentralization of DOT, patients were able to go back to their work after taking treatment under direct observation and henceforth recover in the early stage of treatment. These findings support the progress on achievement on one of the goal of RNTCP, that is, patient should not incur any money for diagnosis and treatment. Another important finding from this study was that majority did not incur any medical costs during treatment, which indicates that free medical services under government are reaching the people.

However, a considerable proportion of patients incurred nonmedical and indirect costs in the intensive phase of treatment. The total costs as percentage of annual family income in this study are much less compared to that published in study by Rajeswari et al. [13] in India and in Thailand study done prior to DOTS where the total costs as percentage of annual family income was 40% and 15%, respectively [24]. However this proportion was more for patients belonging to low economic strata as compared to high economic strata. This highlights that despite the country's propoor health DOTS implementation, they cannot protect households from all costs which necessitates appropriate support security measures for TB patients by governmental and nongovernmental organizations.

The overall mean total cost was significantly high among males (Rs. 3270, *P* < 0.01) and employed (Rs. 3945, *P* < 0.01). This is in conformity to the finding that the economically active age group (15 to 54) patients incur higher costs. The mean total cost was significantly more for extrapulmonary TB patients (Rs. 3915) as compared to pulmonary patients (Rs. 2951). This could be due to the fact that diagnosis of extrapulmonary TB is more difficult as compared to pulmonary TB because it requires tissue diagnosis such as FNAC, biopsy and investigations such as X-ray and CT scan the costs of which are higher. Program needs to provide adequate and easily accessible facilities for diagnosing extrapulmonary TB cases.

The present study, which covered all the ten zones of Chennai Corporation, is representative of the group of TB patients in an urban area. While previous studies on socioeconomic impact of tuberculosis have considered only adult sputum positive patients [13], the present study has included the all age groups and both pulmonary and extrapulmonary TB patients and has given a comprehensive picture of the costs of a cross-section of TB patients registered with the urban TB control program.

Some of the limitations identified are that there could have been recall bias in answering questions on costs. This study has not taken into consideration TB patients who take treatment in the private health sector or the patients taking DOT in rural areas.

## 5. Conclusions

Our study findings strongly suggest that RNTCP has proven to be a cost effective health intervention, with reference to reducing out-of-pocket expenses and indirect costs which indicate that making them return early to work, which in turn benefits their families and in the broader perspective contributes to the overall economic and social development of their country. Reducing out-of-pocket costs to patients may increase the access to the poor people and thus promoting the universal access of TB care services as well. Still, there is a need to provide adequate financial and social support measures to TB patients as two-thirds of TB patients registered in the programme were from the poorer sections of the community. Already attempts have been made to involve private sector, and nongovernmental organisations in the RNTCP. But this study highlights that this activity should be substantially increased. Advocacy, communication, and social mobilization (ACSM) activities on availability of free TB diagnosis and treatment also need to be strengthened to reduce these pretreatment costs.

## Figures and Tables

**Table 1:** Overall mean direct (medical and nonmedical), indirect, and total costs according to patient demographic and socioeconomic characteristics.

Patient characteristics		Direct costs							Indirect costs		Total costs
	No	Medical		Nonmedical		Total direct					
		Mean	SD	Mean	SD	Mean	SD	Mean	SD	Mean	SD
Age											
≤54	260	789	276.60	312	175.57	1101	324.71	2215	450.14	3316	537.87
55+	40	615	228.13	264	157.03	879	261.89	1652	311.40	2532	304.84
Sex											
Male	186	711	261.50	272	163.71	983	305.13	2737	483.14	3720*	545.53
Female	114	855	281.22	360	186.72	1215	329.47	1165	323.41	2380	433.95
Education											
Above higher secondary	51	1117	295.36	306	169.60	1423	320.31	1415	319.75	2838	354.79
Up to high school	117	606	239.71	281	165.61	887	286.31	2261	427.09	3148	479.69
Up to primary	132	771	269.44	328	178.84	1099	317.41	2312	436.70	3411	502.95
Occupation											
Employed	186	731	265.00	278	165.48	1010	309.06	2935	497.18	3945*	557.52
Unemployed	114	821	276.02	351	184.44	1172	324.27	842	279.25	2014	407.21
Per capita income											
Rs. < 1100	74	686	249.48	307	171.54	993	293.21	1851	372.56	2844	418.45
Rs. 1100–2250	158	797	275.10	324	178.14	1121	322.72	2310	444.10	3431	518.26
Rs. > 2250	68	779	262.63	262	158.72	1041	296.92	2058	378.83	3099	410.69
Family size											
≤4	196	785	274.51	304	173.00	1089	320.70	2242	445.59	3331	525.82
>4	104	729	260.37	309	173.15	1038	305.68	1947	397.81	2985	461.33
Family type											
Nuclear	214	758	270.40	310	174.79	1068	318.54	2217	445.79	3286	527.39
Others	86	785	267.09	294	168.51	1079	307.18	1946	388.03	3025	442.83
Type of TB											
Pulmonary	219	573	236.22	223	148.57	796	276.96	2155	440.79	2951	505.31
Extrapulmonary	81	1286	328.91	531	222.75	1817	375.42	2098	394.28	3915*	449.75

^∗^ *P* < 0.01.

**Table 2:** Direct, indirect, and total costs incurred by patients during different phases of treatment (*n* = 300).

Phases of treatment	Direct costs							Indirect costs		Total costs
	Medical		Nonmedical costs		Direct costs					
	Mean	SD	Mean	SD	Mean	SD	Mean	SD	Mean	SD
Before treatment	743	269.18	219	147.45	961	304.99	445	209.4	1407	366.20
Intensive phase	5	22.36	60	77.38	65	80.54	949	303.1	1014	313.01
Continuation phase	18	42.41	27	51.94	45	67.03	745	269.5	790	277.34
During treatment	23	47.94	87	93.14	110	104.69	1694	399.8	1804	411.77

Total costs	765	273.04	306	174.03	1071	321.37	2140	445.8	3211	535.47

**Table 3:** Pretreatment costs to patients with TB.

Types of cost	Nil cost No (%)	Costs incurred by patients		
		Up to Rs. 500 No (%)	Rs. > 500 No (%)	Total No (%)
Medical costs	55 (18.3)	130 (43.3)	115 (38.4)	145 (81.7)
Nonmedical costs	21 (7.0 )	242 (80.7)	37 (12.3)	279 (93.0)
Direct costs	5 (1.7)	147 (49.0)	149 (49.3)	295 (98.3)
Indirect costs	237 (79.0)	6 (2.0)	57 (19.0)	63 (21.0)

Total costs	4 (1.4)	124 (41.3)	172 (57.3)	196 (98.6)

**Table 4:** Percentage of patients who incurred costs during intensive phase of treatment.

Types of cost	Nil cost No (%)	Costs incurred by patients		
		Up to Rs. 500 No (%)	Rs. > 500 No (%)	Total No (%)
Medical costs	293 (97.7)	7 (2.3)	0	7 (2.3)
Non-medical costs	174 (58.0)	121 (40.3)	5 (1.7)	126 (42.0)
Direct costs	173 (57.7)	121 (40.3)	6 (2.0)	127 (42.3)
Indirect costs	226 (75.3)	5 (1.7)	69 (23.0)	71 (24.7)

Total costs	135 (45.0)	91 (30.3)	74 (24.7)	165 (55.0)
